# Dynamic Bayesian network modeling for longitudinal data on child undernutrition in Ethiopia (2002–2016)

**DOI:** 10.3389/fpubh.2024.1399094

**Published:** 2024-12-12

**Authors:** Getnet Bogale Begashaw, Temesgen Zewotir, Haile Mekonnen Fenta

**Affiliations:** ^1^Department of Statistics, College of Science, Bahir Dar University, Bahir Dar, Ethiopia; ^2^Department of Data Science, College of Natural and Computational Science, Debre Berhan University, Debre Berhan, Ethiopia; ^3^School of Mathematics, Statistics and Computer Science, College of Agriculture, Engineering and Science, University of KwaZulu-Natal, Durban, South Africa; ^4^Center for Environmental and Respiratory Health Research (CERH), Research Unit of Population Health, University of Oulu, Oulu, Finland; ^5^Biocenter Oulu, University of Oulu, Oulu, Finland

**Keywords:** Bayes server, counterfactual prediction, conditional probability, maximum posterior, young Lives data

## Abstract

**Introduction:**

Dynamic Bayesian networks improve the modeling of complex systems by incorporating continuous probabilistic relationships between covariates that change over time. This study aimed to analyze the complex causal links contributing to child undernutrition using dynamic Bayesian network modeling, examining both the best- and worst-case scenarios. The Young Cohort of the Ethiopian Young Lives dataset from 2002–2016 was used to analyze the complex relationships among various covariates influencing child undernutrition. We used a built-in Bayes server tool to identify potential features, followed by building the structure of the directed acyclic graph using a structural learning algorithm. The maximum posterior is determined using the relevance tree algorithm. The node with the highest values of mutual information and target entropy reduction, along with the lowest value of target entropy, was considered to have the strongest predictive power in the dataset.

**Results:**

This study revealed that long-term participation in programs increased the likelihood of children being in a normal nutritional state. Key factors influencing the nutritional status of children under two years of age include the mother’s education level, her subjective well-being, and the household’s wealth quintile. Children with educated parents were more likely to have a healthy nutritional status. Additionally, the causal pathway of intervention programs → wealth quintile → child nutritional status consistently exceeded 90% in Waves 3, 4, and 5, indicating a strong relationship. Similarly, the relationship between intervention programs → food security → child nutritional status was nearly perfect at 99.99% in Waves 4 and 5, indicating a strong association. Finally, the study revealed that household participation in intervention programs significantly reduces undernutrition in best-case scenarios, while the absence of support poses a higher risk in worst-case conditions.

**Conclusion:**

The comprehensive intervention program strongly improved household wealth, food security, and maternal well-being, which in turn affected children’s nutritional status.

## Introduction

Child undernutrition is a pressing health concern that affects children, necessitating thorough examination and effective interventions (1, 2). In Ethiopia, child undernutrition is one of the countries where it poses a health challenge (3). In addition to Ethiopia, India, Nigeria, Pakistan, Bangladesh, Indonesia and the Democratic Republic of the Congo are among the seven nations with the highest prevalence of child undernutrition (2, 4, 5). These countries face challenges in undernutrition, including access to nourished food and inadequate healthcare services.

Multivariate logistic regression models are commonly used in studies on child undernutrition outcomes and risk factors (6, 7). However, these models have limitations owing to their static nature and inability to capture temporal relationships (8, 9). The studies conducted by Egata et al. (10) and Bahru et al. (11) utilized a longitudinal model because it is preferable to address this gap by considering within-subject correlations and temporal changes (12, 13). Although longitudinal analysis may not explicitly model transitions between states, it can still provide valuable insights into how variables evolve over time and how they are related to each other. Likewise, Jeyaseelan et al. (14), Owoeye et al. (15) and Begashaw et al. (57) used Markov chain models to address this limitation by incorporating state transitions. However, these models also have limitations because of their assumption of memorylessness, which means that the future state depends only on the current state and not on previous states. Moreover, the research conducted by Hoddinott et al. (16) and Pérez Albertos (17) using the difference-in-differences (DiD) model does not consider the long-term impact of the Productive Safety Net Program (PSNP). On the other hand, Bahru’s et al. (18) marginal structural model has limitations, such as unmeasured time-varying confounding variables and model specification sensitivity.

To address these methodological gaps, this study employs a dynamic Bayesian network (DBN) model that allows for the consideration of uncertainties in the relationships between interventions, covariates, time slices, and nutritional status. Unlike traditional statistical methodologies, which often assume linear relationships or require strong parametric assumptions, DBNs can capture the nonlinear and dynamic relationships among variables. This makes DBNs well suited for analyzing the complex interactions of covariates influencing child undernutrition in a complex socioeconomic context such as Ethiopia (19, 20).

A DBN is a probabilistic reasoning graphical model that represents temporal dependencies between variables in a system (21, 22). It extends traditional Bayesian networks to capture evolving dynamics and is an integral part of artificial intelligence (AI), with applications in finance, healthcare, and other domains that require modeling and predicting complex systems (23, 24). This study provides valuable insights for policymakers and stakeholders by identifying potential interactions between variables to address the challenges related to system dynamics and decision-making. Additionally, DBNs allow the integration of prior knowledge and expert opinions, thereby enhancing the robustness of the modeling process (25).

Currently, DBNs are the most effective models for encoding causal relationships and reasoning uncertain knowledge (26, 27). However, despite Ethiopia having one of the highest rates of undernourished children, DBN use in this context remains relatively underexplored. Our study aimed to fill this gap by applying DBNs to analyze the factors contributing to undernutrition among children in Ethiopia. Identifying key causal pathways and assessing their interactions to improve targeted interventions and policy responses. We also examined which program combinations yielded the most effective improvements and how these changes aligned with the nutritional status of children within participating households.

Many studies on child undernutrition have used composite indices or classified children into categories, such as normal, underweight, stunted, and wasted, or by severity (mild, moderate, and severe). However, these studies often fail to account for concurrent nutritional outcomes, as children may experience multiple forms of undernutrition simultaneously. To address this limitation, we employed a DBN that captures the probability of children being in multiple states of undernutrition over time. This method allows us to analyze the complex interrelationships among various nutritional outcomes and identify the dynamic factors influencing child nutrition. By doing so, we gained deeper insights into how different risk factors and intervention programs interact, ultimately informing more effective strategies to combat undernutrition.

To the best of our knowledge, there is limited evidence regarding the use of the DBN approach to assess the concurrent nutritional status in Ethiopia. Consequently, this proof-of-concept study seeks to explore the potential of DBNs in predicting causal relationships among various parental, household, and child-level nodes/covariates among Ethiopian children under 15 years of age. Additionally, this study aims to identify the strength of key causal relationships contributing to low levels of undernutrition in the best-case conditions, as well as those leading to high levels of undernutrition in the worst-case conditions, counterfactual scenarios in Ethiopia.

## Methods

### Data source and survey design

This study utilized longitudinal data from Ethiopia’s Young Lives of Young cohort (YLCS), an international initiative aimed at addressing childhood poverty and health. The cohort includes approximately 1999 children aged 1–15 (28, 29). The country is highly heterogeneous, with large socioeconomic differences across regional states and between urban and rural areas (30). The surveys were conducted in 20 sentinels across five Ethiopian regions: Amhara, Oromia, Southern Nations, Nationalities, and Peoples (SNNP), Tigray, and Addis Ababa City Administration (CA) from 2002 to 2016 with five waves. Notably, the Productive Safety Net Program (PSNP) operates in 14 sentinels in four regions (excluding Addis Ababa CA), targeting the pro-poor population (31), while the Emergency Aid Programme (EAP) and Health Extension Programme (HEP) operate in all five regions, targeting disadvantaged socioeconomic groups and offering antenatal care, childhood disease management, and micronutrient supplement coverage (32). The study conducted interviews with randomly selected households to determine whether they had participated in the PSNP, EAP, and/or HEP programs within the past 12 months, facilitating the identification of beneficiaries. In the PSNP and EAP, households were categorized as beneficiaries or non-beneficiaries beginning in 2009 (third wave), with the HEP categorization starting in 2013 (fourth wave). These intervention programs were consolidated into a single package using the “program participation status” variable with eight categories (C, P, E, H, PE, PH, EH, and PEH), as detailed in Supplementary Table S1. Similarly, as outlined in Supplementary Table S2, children’s anthropometric conditions encompassed eight distinct states: N, U, S, W, US, UW, SW, and USW (57).

This study did not require ethics approval, as it involved a secondary analysis of publicly available anonymized data. There was no direct interaction with human participants; therefore, informed consent and institutional review board (IRB) approval were not required.

### Data preprocessing pipeline: techniques for anomalies, missing values, and quantization

This study utilizes Bayes Server software, which offers built-in tools for handling missing values, anomaly detection, and feature relevance assessments. It estimates missing values using observed data and probabilistic relationships in the network, without imputing them with static values. Bayes Server employs inference algorithms for temporal models, considering data from past and future time slices. It also uses a probabilistic anomaly detection algorithm to identify outliers or unusual data points, ensuring the quality and reliability of the dataset.

**Figure 1:**
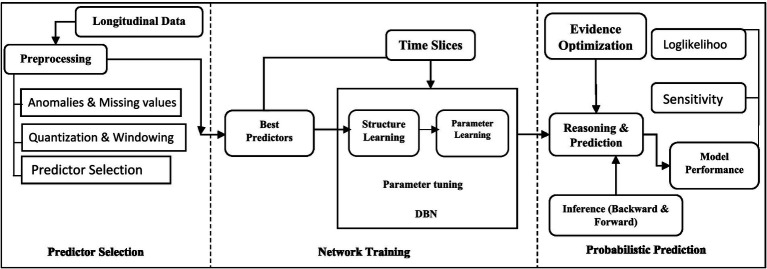
Technical workflow of DBN in application with children nutritional status in YLCS data.

Quantization is essential for preparing continuous variables for analysis in DBNs, as it simplifies modeling and improves interpretability. A balance is needed between using a few categories to prevent overfitting and enough detail to capture variability. Furthermore, the study uses a uniform time interval approach, with data collected every 3.5 years (2002, 2006, 2009, 2013, and 2016) (29, 33). A Lag-1 time window assesses past conditions’ impact on current outcomes, while a Lag-2 window captures longer-term dependencies, enhancing model accuracy and providing deeper insights. As illustrated in Figure 1, our DBN model follows a step-by-step workflow for analyzing children’s nutritional status using YLCS data (see Supplementary material for details).

### Nodes selection in the child nutritional status network

Feature selection is a crucial step in the construction of a DBN. Including all variables in a temporal node can increase the complexity of the network, resulting in higher computational costs and potential convergence issues. Therefore, dedicating extra effort to feature selection is considered to be the best practice. The Bayes Server also offers a built-in feature selection tool that can automatically determine relevant features through the “Add nodes from the data” functionality. Additionally, it uses mutual information and target entropy reduction to assess the conditional dependencies between the features and target variable. The features with the highest mutual information values and target entropy reduction were the most important factors for the predictive performance of the model. As a result, out of the 39 covariates considered, 13 variables were selected as important, reducing model complexity and improving computational efficiency without compromising predictive accuracy (see Supplementary Table S3). The final 13 covariates were treated as nodes in the DBN analysis, allowing for the modeling of temporal dependencies and causal relationships among influential variables (Table 1).

**Table 1:** Description and abbreviations of nodes in the child nutritional status network.

Node category	Abbreviation	Node name	Time-dependent
Time step	*t*	Round of Survey ( t=0 to t=4 )	–
Target state	CNS	Child nutritional status	Yes
Child level nodes	CA	Children age	Yes
	CS	Children sex	No
Parents’s level nodes	MSW	Mother’s subjective wellbeing	Yes
	DA	Father’s education level	Yes
	DE	Father’s age	Yes
	MA	Mother’s age	Yes
	ME	Mother’ education level	Yes
Household level nodes	HS	Household size	Yes
	WQ	Wealth quintile	Yes
	FS	Food Security	Yes
	HHA	Household head age	Yes
	HHS	Household head sex	Yes
Intervention node	PS	Program Participation Status	Yes

t (recommended approach in Bayes server as shorthand for time) represents time, where t=4 indicates time step 4. All time steps are zero-based, meaning t=0 is the initial time step.

### Structure learning

This study created a directed acyclic graph (DAG) structure to analyze child undernutrition data. The process involved consulting experts, reviewing the literature, and identifying key nodes. An initial DAG structure was constructed, defining nodes representing factors related to child undernutrition and hypothesizing directional relationships. The DAG structure was then refined using structure-learning algorithms in Bayes Server Desktop version 10.9. However, several erroneous links were produced, requiring validation and correction, with certain causal relationships, such as those from DA → HHS, MSW and ME → CS, CS → WQ, and CA → MA, identified as implausible. These causal links are static and cannot be influenced by other variables, and were confirmed during the structural learning process.

**Figure 2:**
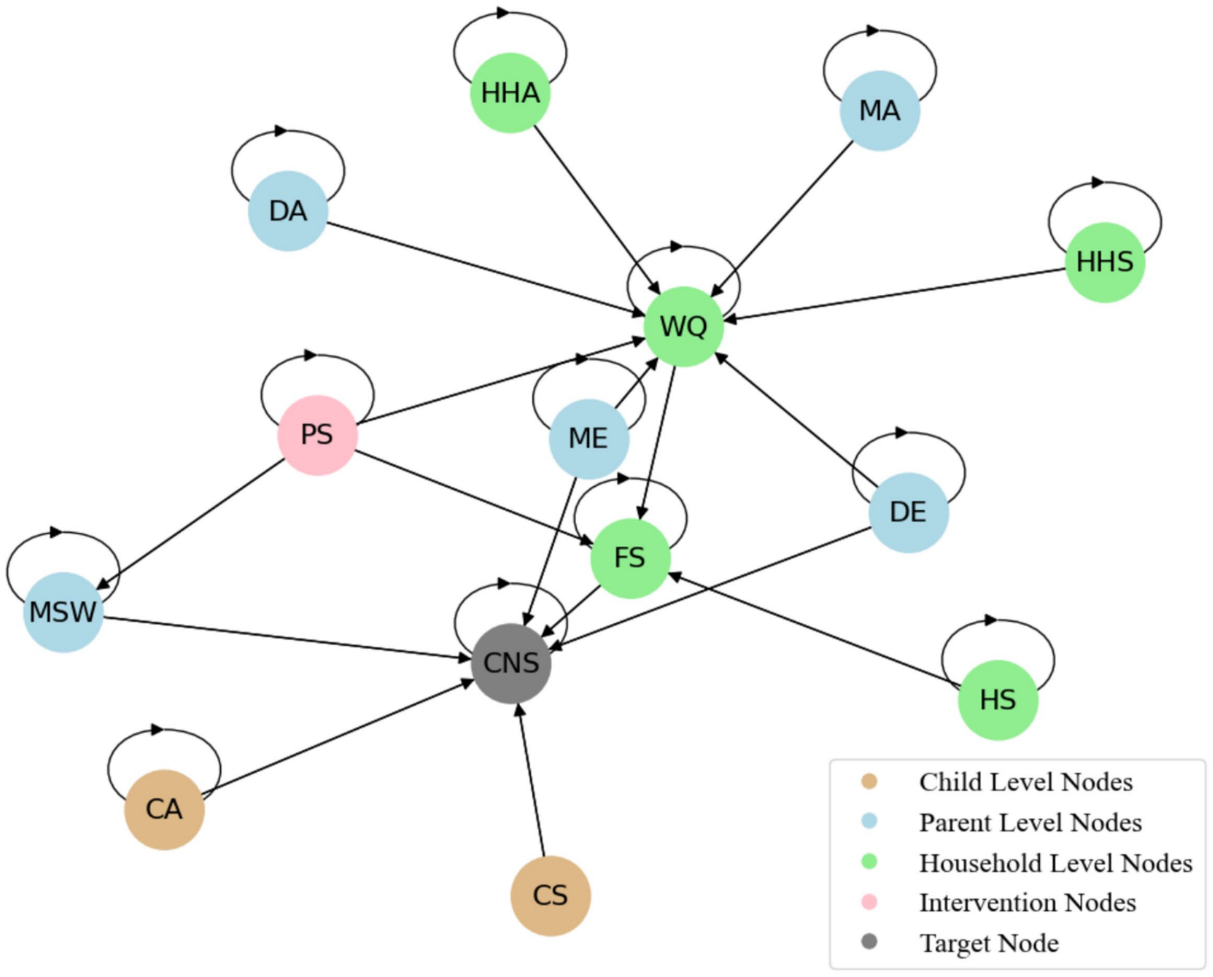
Temporal causal pathways in DAGs for child undernutrition. NB: The detailed DAG causal pathways are listed in Supplementary Table S5 because the number of causal pathways across time points is extensive and difficult to display in a single DAG structure.

The final DAG structure was validated and refined to ensure consistency with the domain knowledge and plausibility of the generated links, accurately representing child undernutrition dynamics while considering system complexity (Figure 2). In the DAG, the child’s sex is the only node that does not have a curved arrow pointing back. This means that all other nodes, excluding the child’s sex, have a temporal dependency on their responses (Supplementary Table S5). In addition, all temporal nodes except the PS and FS nodes had five time slices. The PS and FS nodes had only three time slices, with zero placeholder values used for the first and second visits to account for the missing data (Supplementary Table S4). The fundamental concepts of temporal relationships and causal dependencies in DBNs are briefly discussed in the Supplementary material.

### Statistical analysis

DBNs have expanded the scope of traditional BNs to include temporal features such as changes in child undernutrition data over discrete time intervals (t = 1, 2, 3, …, T). The set of variables in a DBN is represented by $X=\left\{X_{1},X_{2},\ldots ,X_{N}\right\}$ , with conditional dependencies represented by directed edges. The transition model in a DBN is defined by the joint probability distribution over time (34, 35).


$$P\left(X_{t}|X_{t-1},\ldots ,X_{t-T}\right)=\overset{N}{\underset{i=1}{\prod }}P\left(X_{t,i}|\mathrm{Pa}\left(X_{t,i}\right)\right)$$

where, $P\left(X_{t}|X_{t-1},\ldots ,X_{t-T}\right)$ is the joint probability distribution of the state variables at time t given the past observations, $X_{t}$ represents the state of variables at time t , and the conditional dependencies capture how the state at the current time depends on the previous states, $X_{t,i}$ is the state of node i at time t, and $\mathrm{Pa}\left(X_{t,i}\right)$ represents the parents of node i at time t in the graph (Figure 2).

The model incorporates the transition probabilities between time steps t−1 and t . For example, the probability of transitioning from state $X_{\left(t-1,\;i\right)}\;to\;X_{t,i}$ can be represented as:


$$P\left(X_{\left(t,i\right)}|X_{\left(t-1,i\right)}\right)$$

The model parameters were calibrated against available data using maximum likelihood estimation (MLE). The log-likelihood function for the entire dataset D can be defined as:


$$L\left(\Theta ;D\right)=\overset{5}{\underset{t=1}{\sum }}\overset{n}{\underset{i=1}{\sum }}\log P\left(X_{t,i}|\mathrm{Pa}\left(X_{t,i};\Theta \right)\right)$$

where Θ represents the parameters of the DBN and D is the observed data.

The structure of the DBN was learned from data using the Peter and Clark (PC) algorithm. Let D be the dataset; the objective is to find the optimal structure G that maximizes the likelihood of the data:


$$G_{\mathrm{optimal}}={\mathrm{argmax}}_{G}P\left(D|G\right)$$

The PC algorithm efficiently explores possible network structures by systematically identifying conditional independence relationships among data variables, which helps to determine the structure of the DBN. The PC algorithm constructs a DAG starting from an empty graph by adding edges based on a statistical test of conditional independence (36, 37). It constructs a partially directed acyclic graph (PDAG) and applies rules such as the “V-structure” and “immorality” rules to obtain a full DAG.

The DBN parameters ( θ ), including conditional probability tables, were estimated from the data D using the following mathematical formula:


$$\theta_{\mathrm{optimal}}={\mathrm{argmax}}_{\theta }P\left(D|\theta ,G_{\mathrm{optimal}}\right)$$

This step involves estimating the parameters that maximize the likelihood of the data, given the chosen DBN structure.

Maximum *a posteriori* (MAP) queries, also known as the most likely explanation (MPE), aim to determine the most likely state of the target variables based on the evidence observed in the data. MAP estimation in DBNs involves finding the sequence of hidden states that maximizes the posterior probability, given the observed evidence. This can be formulated using Bayes’ theorem as follows:


$$\mathrm{argma}x_{x}P\left(X_{t}|E_{1:t}\right)=\mathrm{argma}x_{x}\frac{P\left(E_{1:t}|X_{t}\right)P\left(X_{t}\right)}{P\left(E_{1:t}\right)}$$

where:

$X_{t}$ : represents the hidden state at time t; $E_{1:t}$ denotes the evidence observed up to time t; $P\left(X_{t}|E_{1:t}\right)$ is the posterior probability of the hidden state given the evidence; $P\left(E_{1:t}|X_{t}\right)$ is the likelihood of observing evidence given the hidden state; $P\left(X_{t}\right)$ is the prior probability of the hidden state; and $P\left(E_{1:t}\right)$ is the marginal likelihood of the evidence.

The relevance tree algorithm was utilized to compute the MAP estimate, which efficiently found the most likely sequence of hidden states based on model parameters and observed data. This algorithm is particularly useful for computing probabilities in DBNs with large state spaces and complex variables because it constructs a tree structure.

In causal inference, we model counterfactual outcomes to estimate the probability of an event under hypothetical conditions. Let Y represent the outcome of interest, such as child nutritional status, and let $X=\left\{X_{1},X_{2},\ldots ,X_{n}\right\}$ be a set of covariates, including factors like household, caregiver, and parental information. To formalize counterfactual reasoning, we typically use a causal graph or a Bayesian network, where directed edges represent the causal dependencies between variables.

In this framework, the counterfactual probability can be expressed as:


$$P\left(Y^{cf}=y|\mathrm{do}\left(X^{cf}=x\right)\right)$$

Here, $Y^{cf}$ refers to the counterfactual outcome of Y , representing the value that would have been observed if the hypothetical conditions $X^{cf}=x$ were true. In contrast, Y is the observed outcome under normal conditions. Similarly, $X^{cf}=x$ refers to a set of counterfactual conditions, which represent a hypothetical intervention or modification of the covariates. The operator, $\mathrm{do}\left(X^{cf}=x\right)$ denotes an intervention where we actively set $X^{cf}=x$ , effectively modifying the system. Therefore, the expression $P\left(Y^{cf}=y|\mathrm{do}\left(X^{cf}=x\right)\right)$ gives the counterfactual probability of Y=y if the intervention $X^{cf}=x$ had been applied. For instance, if we consider X to represent maternal education level and Y as child nutritional status, the counterfactual probability $P\left(Y^{cf}=y|\mathrm{do}\left(X^{cf}=x\right)\right)$ quantifies the likelihood of different nutritional outcomes had the maternal education level been fixed at x, regardless of other influencing factors.

### Model performance metrics

Model performance was assessed using three key metrics: log-likelihood and Value of Information (VOI). The log-likelihood metric assesses how well the model fits the observed data and provides an indication of its accuracy. Finally, VOI measures the potential value of acquiring additional information to improve the model’s predictions.

### Computational environment

The study evaluated various software options, including GeNIe and SMILE (38), Hugin Expert (39), Netica (40), R (bnlearn and gRain packages) (41–43), and BayesiaLab (44). Bayes Server Desktop version 10.9 was found to be most effective for managing the complexities of a DBN model with numerous variables. It enabled precise inference, updated beliefs, and assessed uncertainty in child nutrition decisions. The intuitive graphical interface and efficient analysis were achieved on a system with an Intel i7-9300H, NVIDIA GTX 1650 GPU, and 32 GB of RAM.

## Results

### Conditional probabilities of child nutritional status and household characteristics over time

**Figure 3:**
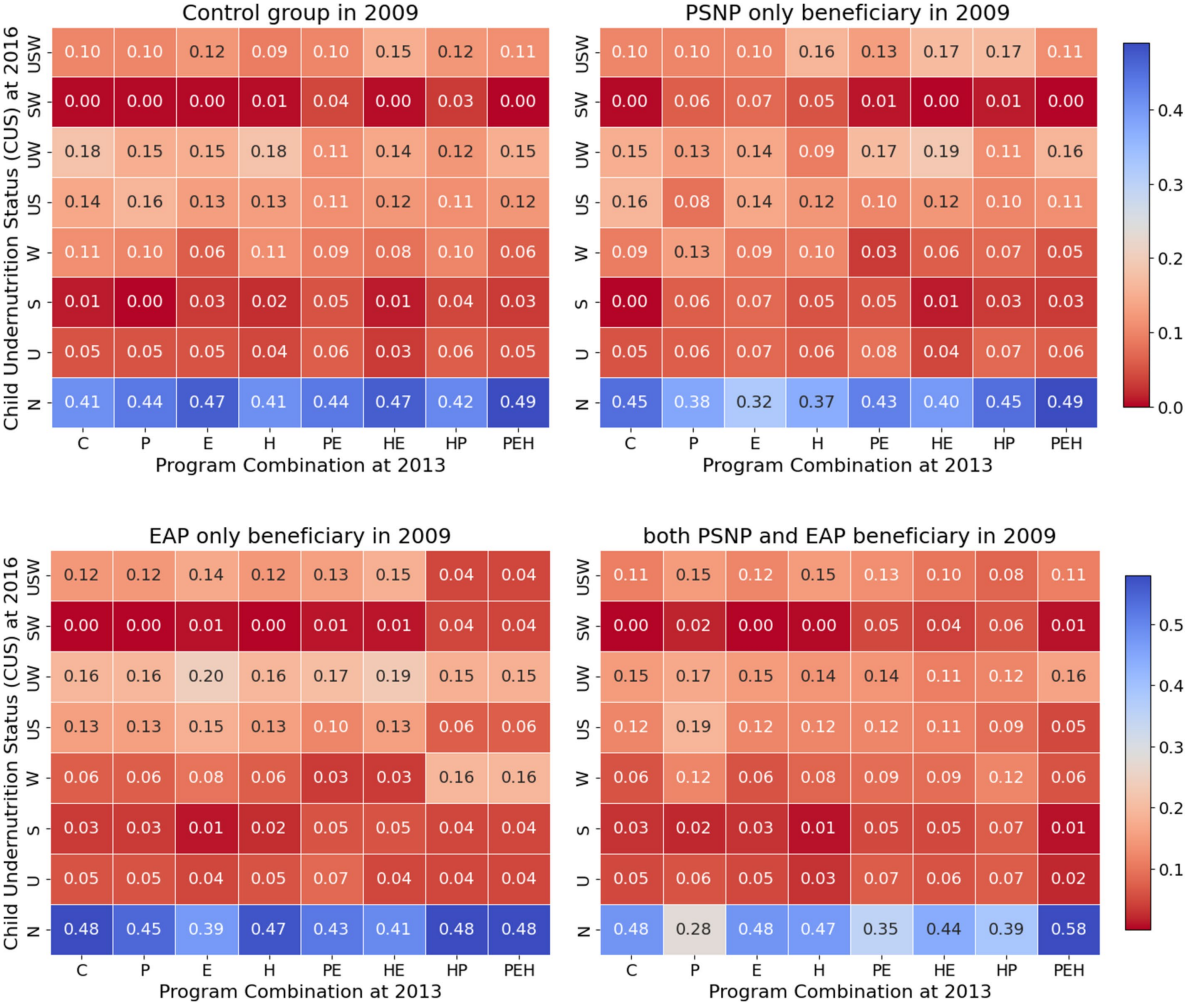
Conditional probability of child nutritional status by household program participation status over time.

In 2016, 41% of the children from households without any program enrollment had a normal nutritional status. In contrast, those enrolled in the PEH in 2013 had a 49% chance of having a normal nutritional status and a 15% chance of being underweight and wasting concurrently (UW). Between 2009 and 2013, in 2016, 58% of the households participating in the PEH and PEH programs were normal children, 16% were underweight, and 16% were wasting children (Figure 3).

**Figure 4:**
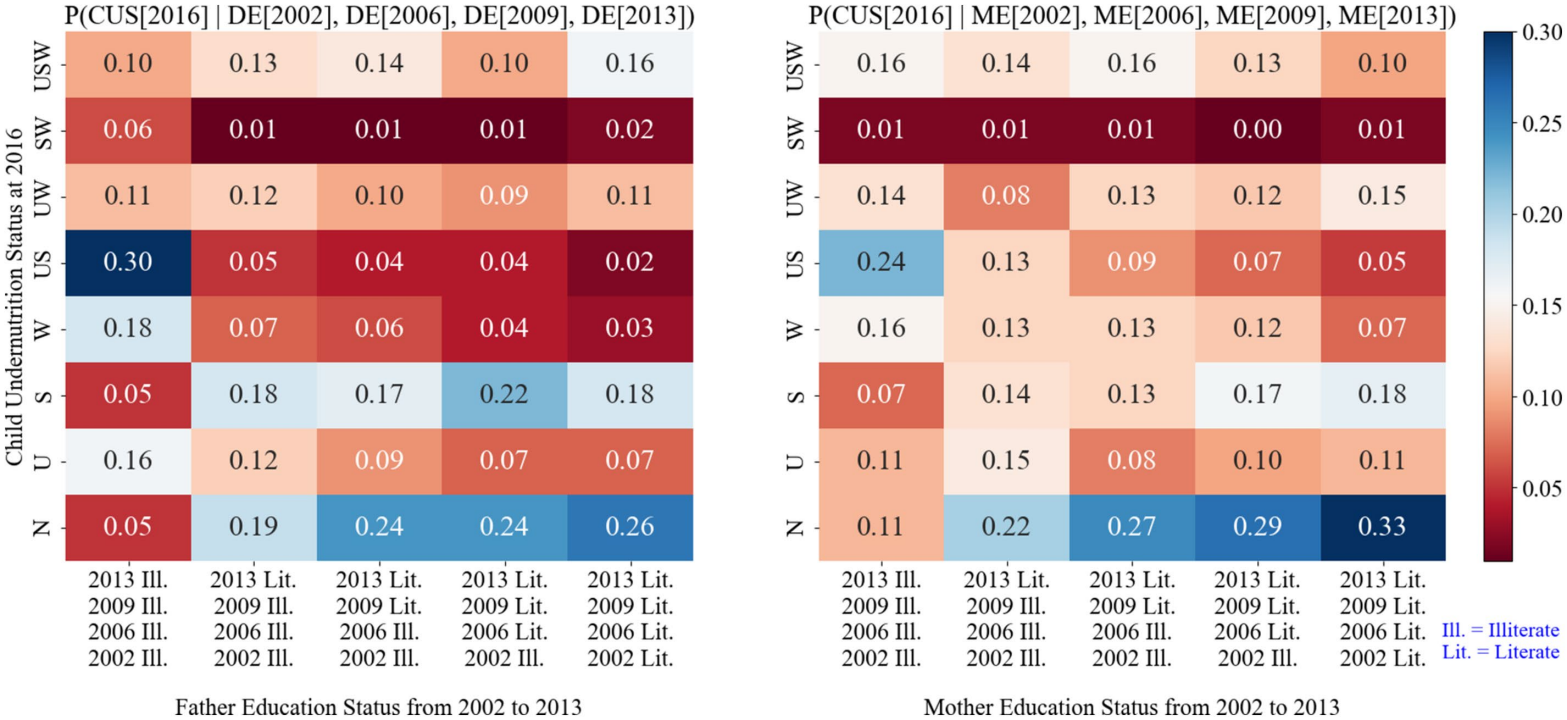
Conditional probability of child nutritional status based on parental education history.

Figure 4 shows the conditional probabilities of children’s undernutrition status based on the parents’ education levels over time. In 2016, children with illiterate parents had a greater risk of developing underweight and stunted conditions concurrently than those with literate parents. The risk was 30% greater for children with illiterate fathers and 26% greater for children with literate mothers.

**Figure 5:**
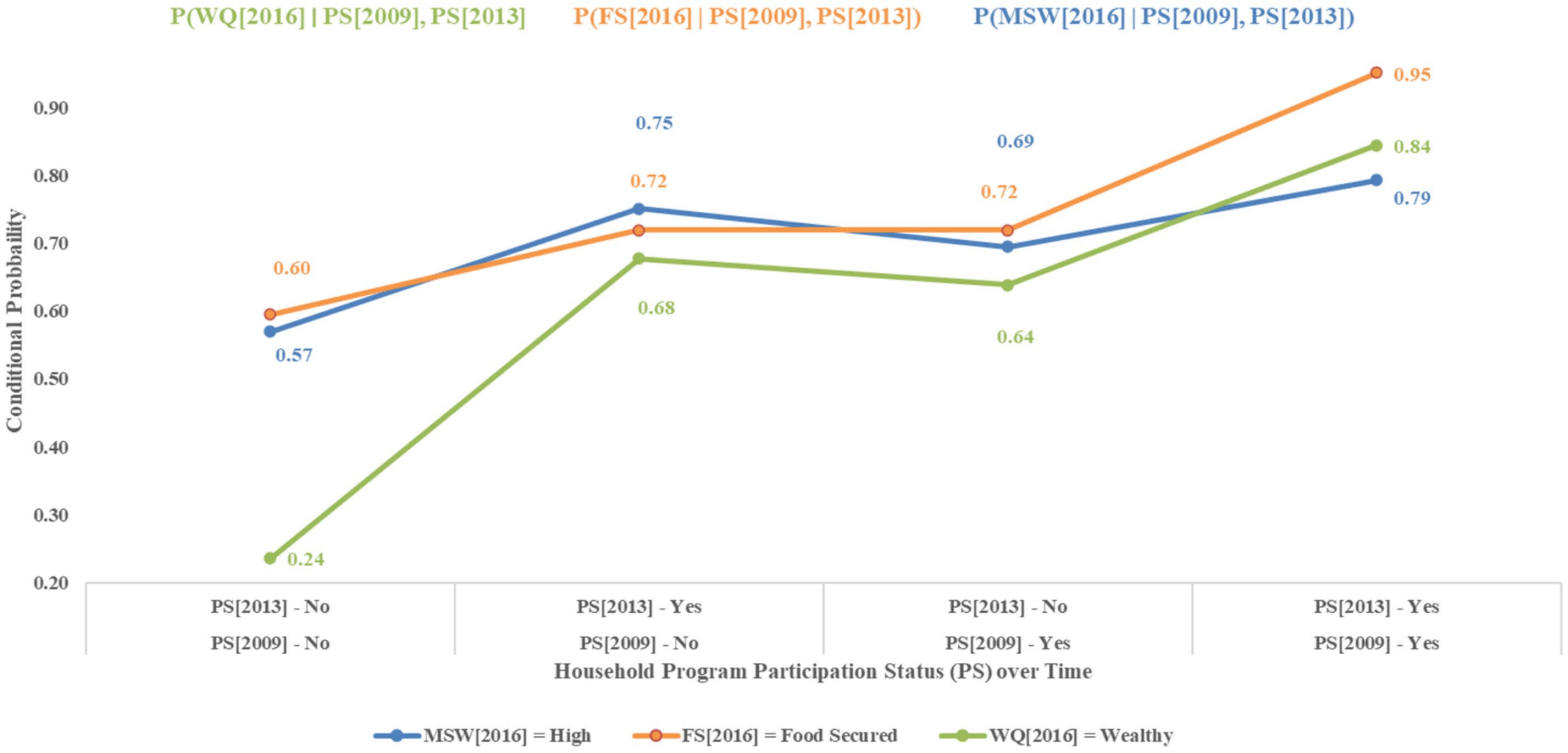
Conditional probabilities of household characteristics over time by programme participation status.

The study showed that in 2016, mothers in households without prior intervention had a 57% chance of improved well-being. For those who joined after 2013, the likelihood increased to 75%. However, the number of households that joined in 2009 but discontinued after 2013 decreased to 69%. Among those engaged since 2009, 79% had significantly increased well-being, indicating that sustained program participation positively impacts mothers’ well-being. In 2016, households that were not in the intervention program had a 60% probability of securing food access. Those participating in 2009 or 2013 had a 72% probability, whereas those participating in both years had a 95% probability. Finally, in 2016, households not previously enrolled in the programme had a 24% probability of being wealthy. For those who received support from 2013 onward, the probability increased to 68%. For households supported beginning in 2009, but discontinued after 2013, the probability was 64%. Households that have been continuously supported since 2009 have an 84% probability of earning wealth (Figure 5).

**Figure 6:**
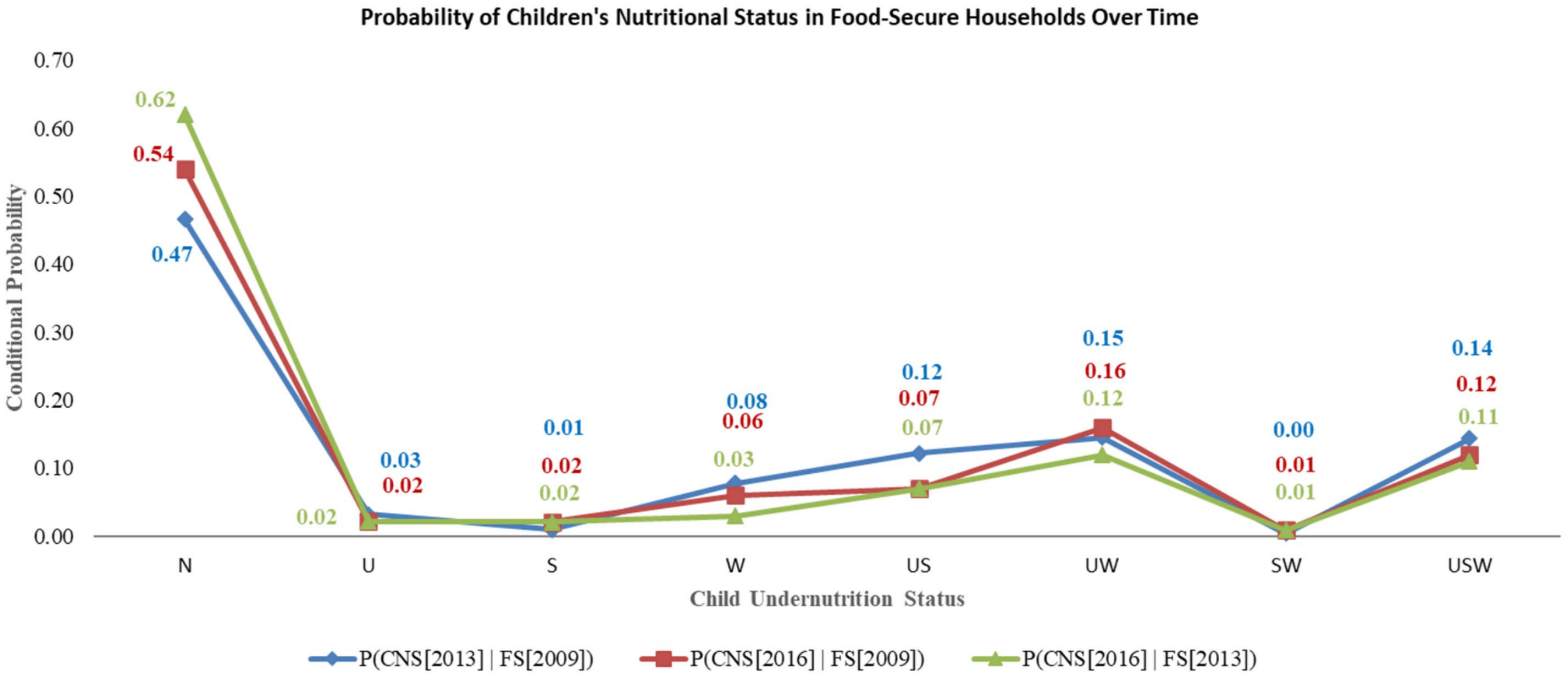
Trends in the conditional probability of child undernutrition in food-secured households.

Moreover, since 2009, the likelihood of a child being classified as having normal nutritional status in 2013 was 47% when she was a family with access to food security. This probability increased to 54% in 2016 for children from households that had gained access to food security in 2013. In contrast, the likelihood of a child experiencing concurrent underweight, stunting, and wasting (USW) in 2013 was 11% among families with continuous access to secure food since 2009. This probability has decreased slightly to 11% in 2016 for a child in a household with sustained food security since 2013 (Figure 6).

### Key household and parental factors influencing food security, well-being, wealth, and child nutrition outcomes

In 2009, households with more than six members in the poor wealth quintile and those participating in PSNP programs were most likely to experience food insecurity. By 2013, households with fewer than six members in the poor wealth quintile and enrolled in both PSNP and EAP programs were most likely to have secure food access. By 2016, households with fewer than six members in the economically wealthy quintile and participating in all three programs (PSNP, EAP, and HEP) were most likely to maintain food security (Table 2).

**Table 2:** Maximum posterior effect of household characteristics on food security.

t	HS	WQ	PS	FS
2009	>6	Poor	P	Insecure
2013	<6	Poor	PE	Secure
2016	<6	Poor	PEH	Secure

In 2002, 2006, and 2009, mothers not enrolled in the intervention program were most likely to have low subjective well-being regardless of their age. However, in 2013 and 2016, mothers under the age of 30 but enrolled in EH and PEH, respectively, were most likely to have high subjective well-being (Table 3).

**Table 3:** Maximum posterior effect of maternal characteristics and program enrollment on subjective well-being.

*t*	MA	PS	MSW
2002	Under 30	–	Low
2006	30–50	–	Low
2009	Under 30	C	Low
2013	Under 30	EH	High
2016	Under 30	PEH	High

Dash (−) indicates that the program was not available in that specific time slice.

In 2002 and 2006, households were most likely to be poor if the mother was illiterate, both parents were aged 18–30, or the household head was male, regardless of the father’s education level and household head’s age. However, in 2009, 2013, and 2016, households headed by males aged 30–50 years and fathers of the same age range were more likely to achieve wealth when enrolled in PE, PEH, or PE programs, respectively (Table 4).

**Table 4:** Maximum posterior effect of household characteristics and program enrollment on wealth status over time.

t	DE	ME	DA	MA	HHA	HHS	PS	WQ
2002	Illiterate	Illiterate	18–30	18–30	18–30	Male	–	Poor
2006	Literate	Illiterate	18–30	18–30	30–50	Male	–	Poor
2009	Literate	Illiterate	30–50	Under 30	30–50	Male	PE	Wealthy
2013	Illiterate	Literate	30–50	30–50	30–50	Male	PEH	Wealthy
2016	Literate	Literate	30–50	30–50	30–50	Male	PE	Wealthy

Dash (−) indicates that the program was not available in that specific time slice.

In 2002, households with illiterate fathers and literate mothers were most likely to have undernourished (US) female infants. By 2006, households with illiterate mothers, literate fathers, and wealthier status were the most likely to have stunted male children. Conversely, in 2009, households with literate mothers and illiterate fathers in poor conditions had a greater chance of being undernourished (SW) preadolescent males. In 2013, households with both literate parents and wealthier status were most likely to have preadolescent females with normal nutritional status. Finally, in 2016, households with literate mothers, illiterate fathers, and wealthier status were most likely to have wasted adolescent females (Table 5).

**Table 5:** Maximum posterior effect of parental education and household wealth on child nutritional status over time.

*t*	ME	DE	WQ	MSW	FS	CA	CS	CNS
2002	Literate	Illiterate	Poor	Low	-	Infants (0–2 years)	Female	US
2006	Illiterate	Literate	Wealthy	Low	-	Early childhood (2–6 years)	Male	S
2009	Literate	Illiterate	Poor	Low	Insecure	Preadolescence (6–12 years)	Male	SW
2013	Literate	Literate	Wealthy	High	Secure	Preadolescence (6–12 years)	Female	N
2016	Literate	Illiterate	Wealthy	High	Secure	Adolescents (12–18 years)	Female	W

Dash (−) indicates that the program was not available in that specific time slice.

### Causal pathway strength over time: evidence from posterior probabilities in the DAG model

Due to the extensive number of causal pathways across time points, displaying them all in a single DAG structure has been challenging. The DAG structure presents detailed causal pathways across time points based on hard evidence, with parent and child nodes and their causal edges indicated by right arrows (Supplementary Table S5). These arrows show the temporal direction of the causal connection, specifying which parent node is connected to a child node at which time slice. Additionally, temporal dependency edges are included, indicating where a node has a connection to itself across time slices. The probabilities indicate edge strength, with higher probabilities indicating stronger relationships or causation.

In this study, we found varying edge probabilities in the relationship from PS → MSW in the posterior DAG across different waves, ranging from 74 to 93%, indicating a consistent strengthening of this connection over time. Mothers in households involved in EH and PEH intervention programs exhibited higher levels of subjective well-being. The edge probabilities for PS → FS and PS → WQ were 100% during waves 2, 3, and 4 as well as during waves 3 and 4. This perfect certainty in the posterior DAG suggests a robust link between household food security and wealth quintiles within combined initiative programs. Specifically, households with fewer than six members who received assistance from both the PSNP and EAP in 2013 were more likely to have secure access to food (Supplementary Table S5).

The study revealed a strong relationship between the wealth quintile and food security, with a near-certain relationship in Waves 2 and 4 (99.99%) and a slightly weaker but still significant relationship in Wave 3 (95%). This finding underscores the substantial impact of wealth quintiles on food security, indicating that individuals in higher-wealth quintiles are more likely to experience better food security than those in lower-wealth quintiles. The relationship between food security and undernutrition was 48% in Wave 2, rising to 100% in both Wave 3 and Wave 4. This finding indicates a strong and enduring link between food security and children’s nutritional status over time.

### Examining child nutrition outcomes in Ethiopia: best- and worst-case situation

The study aimed to find a condition that reduce child undernutrition (best-case situation) in Ethiopia by setting the “Normal” category to 100% and adjusting counterfactual values. The original values for underweight (U), stunted (S), and wasting (W) were 3.8, 7.8, and 5.3%, respectively. For combined undernutrition conditions, the initial values were underweight and wasting (US) at 14.1%, underweight and stunting (UW) at 11.1%, stunting and wasting (SW) at 0.1%, and a combination of all three conditions (USW) at 8.1%. This idealized scenario represents a condition in which no child suffers from undernutrition.

The corresponding counterfactual results under the header “Predicted” are listed in Supplementary Table S6. The up and down arrows next to the predicted value indicate an increase and decrease, respectively, compared with the original value. The results of the best-case counterfactual situations can be interpreted as follows. The results of the best-case counterfactual scenarios reveal that by the time children reach eight years old (time slice 2), households with fewer than six members experience increased counterfactual probabilities of 31.7, 26.2, and 21.2% for time slices 2, 3, and 4, respectively, compared to the original values. This trend suggests that smaller household sizes during early childhood may reduce the likelihood of undernutrition. As household size increases, food insecurity also tends to increase, which in turn increases the likelihood of children experiencing undernutrition. Likewise, counterfactual probabilities for poor maternal subjective well-being decreased as children aged 8 to 15–28.3, 21.4, and 14.3% for time slices 2, 3, and 4, respectively—compared to the original probabilities. Simultaneously, the probabilities for better subjective well-being increased (31.4, 19.7, and 23.1%), highlighting the importance of improving maternal well-being as an effective strategy for reducing child undernutrition. Similarly, the decreased counterfactual probabilities for poor maternal well-being—28.3, 21.4, and 14.3% for time slices 0, 1, and 2, respectively—indicate an improvement as children age to 8 years.

To emphasize the worst-case situation, the probability of a child being categorized as ‘Undernourished’ is set to 100%, which represents a scenario where every child is experiencing some form of undernutrition. Conversely, the probability of children being classified as ‘Normal’ (i.e., not undernourished) is set to 0%. The model shows that a low subjective wellbeing of mothers significantly impacts child undernutrition, with the probability of children being undernourished increasing as the mother’s wellbeing declines. This is particularly evident in the later time slices, where the probability reaches 3.1% for those in high wellbeing and 40.2% for those in low wellbeing. Smaller households (<=6 members) initially have a higher probability of undernutrition (38.2% in Time slice 0), but this decreases over time. Larger households (>6 members) initially experience a smaller proportion of undernutrition, but this increases in later time slices (Supplementary Table S7). Similarly, poor households and those experiencing food insecurity show much higher probabilities of undernutrition across all time slices. In contrast, wealthier households and those with food security experience fluctuations, but they generally see a higher probability of ‘Normal’ status compared to their poorer counterparts. Similarly, illiterate fathers and mothers contribute to a higher likelihood of child undernutrition. However, the scenario shows a complex pattern with some improvements in later time slices, possibly due to interventions or other factors impacting education.

### Impact of intervention programs on combating child undernutrition

**Figure 7:**
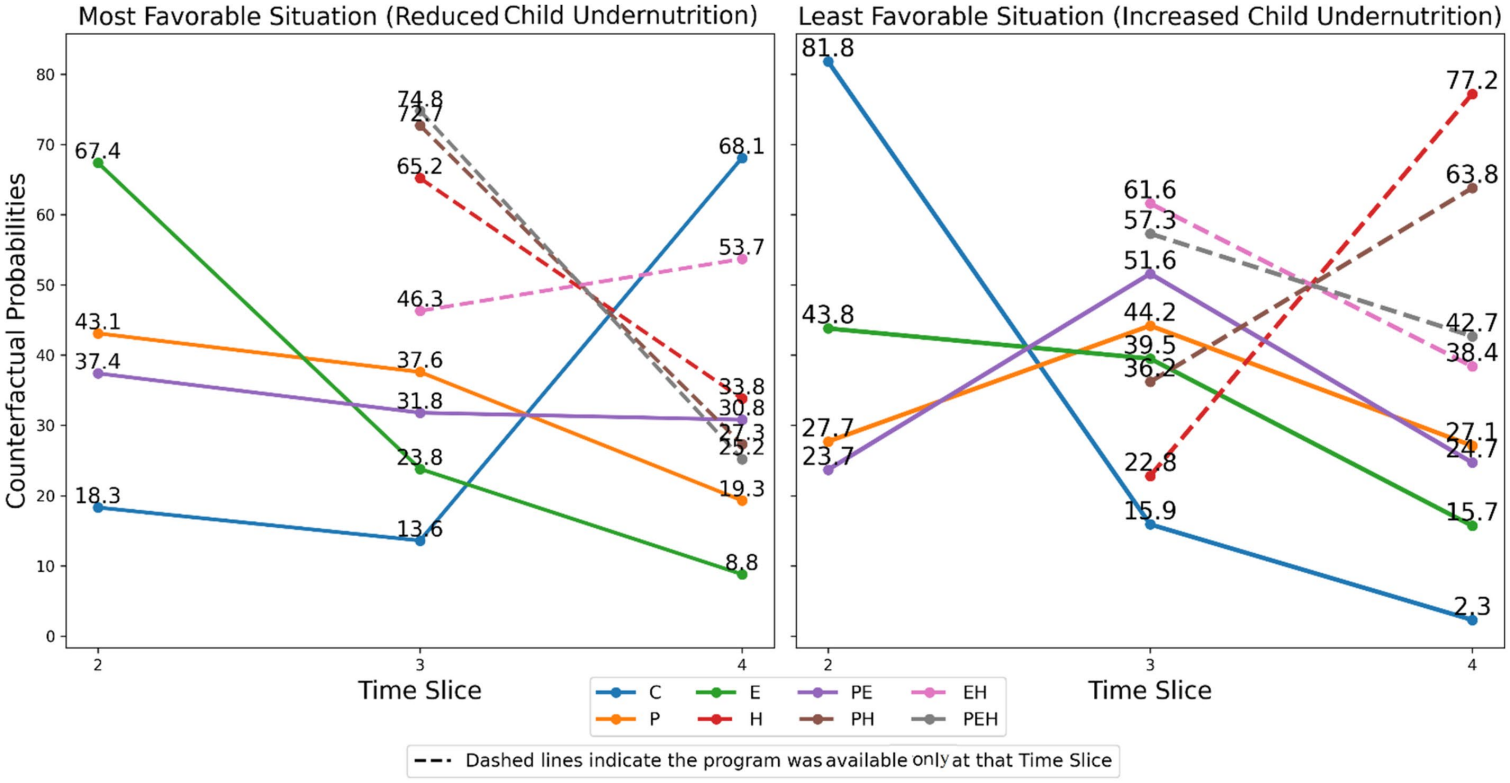
Most- and least favorable situation of child undernutrition in Ethiopia based on household participation in intervention programs.

As shown in Figure 7, the predicted counterfactual probability of household program participation combinations across each time slice is visualized for both the best- and worst-case scenarios. In the most favorable scenario, the probability of program participation (except EH) is highest at time slice 2, followed by time slice 3, and decreases further at time slice 4. In contrast, in the least favorable scenario, the probability shows a decline from time slice 2 to time slice 3, and again from time slice 3 to time slice 4. This suggests that early household participation in the program provides the most effective scenario for improving children’s nutritional status, whereas decreased participation over time correlates with higher likelihoods of child undernutrition.

For non-beneficiary households under the best-case scenario, the predicted probability is lowest at time slice 2 and increases in time slices 3 and 4. Conversely, under the worst-case scenario, this probability is highest at time slice 2 and decreases in subsequent time slices, indicating a greater risk of undernutrition for non-participating households.

### Performance metrics of DBN model

Table 6 shows that variables such as MSW, DE, PS, HS, and MA had higher mutual information values, indicating stronger relationships with child undernutrition within the network. Additionally, variables such as PS, DE, HS, CA, and FS exhibit high target entropy reduction, suggesting their importance in reducing uncertainty within the network. Conversely, covariates such as DE, PS, HS, MA, and MSW exhibited lower target entropy values, implying lower uncertainty and greater predictability of the target variable compared to other covariates in the network (Table 6).

**Table 6:** Predictive performance of nodes in the DBN.

Variables	Mutual information	Target entropy reduction	Target entropy
MSW	0.97	0.44	0.03
DE	0.96	0.52	0.03
PS	0.94	0.68	0.02
HS	0.92	0.51	0.04
MA	0.91	0.45	0.05
ME	0.89	0.48	0.04
WQ	0.88	0.54	0.05
HHA	0.82	0.39	0.04
HHS	0.79	0.42	0.06
CA	0.78	0.52	0.07
DA	0.75	0.37	0.05
FS	0.73	0.57	0.05
CS	0.62	0.54	0.06

## Discussion

Child malnutrition is a complex health problem affected by various factors, such as food security, socioeconomic status, parental education, mother well-being, and access to healthcare services, as indicated in previous research studies (45, 46).

In our study, we found that children with educated parents were more likely to have a normal nutritional status. A recent study by Zhu et al. (47) revealed that socioeconomic factors impact the body mass index (BMI) of both primary and secondary caregivers, which in turn affects the BMI of their children through a causal relationship. Research has demonstrated that the educational level of parents and primary caregivers significantly impacts the nutritional status of children, with improvements in nutritional status observed as education levels increase (48, 49).

Access to nutritious food and essential services such as clean water, electricity, healthcare, and proper sanitation facilities tends to increase as household wealth increases. This improvement in access leads to better nutritional status for children. In this regard, the intervention program showed a significant association with wealth quantiles (PS → WQ), particularly in the 4th and 5th waves, suggesting a positive impact on household wealth improvement. Research indicates that children from economically disadvantaged and displaced families are particularly vulnerable and may require assistance in accessing food and healthcare services (50–52).

The study highlights the substantial impact of mother well-being on child nutrition, attributed to improved mental health, emotional availability, and caregiving practices. Mothers with enrolled households in the program were most likely to report high subjective well-being (PS → MSW). Health workers, through regular nutritional counseling, significantly improve child-feeding practices, reducing undernutrition risk in children. Sunguya et al. (53) showed that health workers’ nutritional training enhances feeding frequency, energy intake, and dietary diversity in children aged six months to two years. Furthermore, this study is supported by a study by Miller et al. (54), which highlighted the effective management of children by health extension workers.

The study showed that the improvement in a household’s food security status is conditional on the combination of the intervention program (PS → WQ), indicating that the intervention program positively impacts household food security. A study by Tadesse and Gebremedhin (55) using propensity score matching found that PSNP significantly enhances the income and food security of households in chronically food-insecure areas by increasing consumption expenditure and daily caloric intake. This finding is further supported by a study conducted by Bahru et al. (18), which also confirmed the positive impact of PSNP on household food security and child meal frequency. Furthermore, a study conducted by Gilligan and Hoddinott (56) using a difference-in-differences matching estimator revealed that households in rural areas affected by drought experienced improvements in food security when they received support from emergency food aid programs.

The DBN model predicts that household participation like in PSNP, EAP, and PE programs can significantly reduce undernutrition in the best-case conditions. However, counterfactual probabilities for non-beneficiary households are higher. These results highlight the impact of program interventions on reducing undernutrition over time, emphasizing the higher risk of undernutrition without support. Early and sustained program engagement is crucial for improving children’s nutritional outcomes. The best-case conditions for reducing childhood undernutrition in households are based on earlier participation in the program (except EH program combination), with the higher predicted probabilities for participation in the intervention program and clearly visualized in Figure 7.

Strong temporal causation is shown in the Supplementary Table S5 for variables such as CNS, PS, FS, and WQ, all of which have a significant effect on child undernutrition in Ethiopia. From time 0 to time 1, the CNS is extremely dependent, however PS shows long-lasting benefits from time 2 to 4. In order to reduce undernutrition, FS is essential for mitigating undernutrition, and WQ highlights the significance of household economic position. Weaker temporal dependencies like household head age and child age indicate that the influence of variables diminishes with time.

### Strengths, limitations, and future work

A significant methodological contribution of this study is the application of DBNs to analyze child undernutrition, a relatively novel approach in public health research. This study demonstrates how DBNs can provide temporal dependencies, capturing how risk factors and nutritional states change and influence each other over different time periods.

To maintain a manageable publication length, we opted not to include the predicted counterfactual probabilities for all seven undernutrition states, as presenting each state individually would be overly bulky. Instead, these states were consolidated into a single “Undernourished” category, for modeling worst-case situation of child undernutrition. Additionally, query outputs for all variables and time steps were excluded, as they were primarily used internally for structure learning, assessing causal pathway, and edge strengths within the DBN. A system with 13 nodes has an order of 2^78^ possible graph structures, as calculated using the formula $\left(2^{\frac{n\left(n-1\right)}{2}}\right)$ . Due to this immense complexity, attempting to visualize all potential graph structures for a system with 16 nodes and 5 time points becomes practically impossible.

For future researchers working with DBNs, access to high-performance computing systems is essential to handle larger models efficiently. Future research could explore hybrid models that integrate additional machine learning techniques and causal artificial intelligence (AI), which are supported in Bayes Server through its API, to capture more complex relationships and enhance causal insights.

## Conclusion

Overall, our DBN model offers a novel approach for understanding child undernutrition in Ethiopia by capturing temporal relationships and identifying critical risk factors. This study demonstrates how DBNs can enhance public health research, providing policymakers and practitioners with a predictive tool for targeted interventions. The combined intervention program showed a strong causal relationship with enhancing the food security, wealth, and subjective well-being of mothers. Wealth quantiles provide better access to nutritious food, healthcare services, and education, which are vital for ensuring optimal growth and development in children. Food security ensures that adequate, safe, and nutritious food for healthy growth is essential for promoting healthy growth in children. Mothers’ subjective well-being, including mental health, stress levels, and overall satisfaction, also influences children’s nutritional status.

The study provides valuable insights for policymakers and health practitioners to simulate “what-if” scenarios to optimize nutritional outcomes and forecast at-risk situations leading to child undernutrition. It highlights high-risk factors like food insecurity, low maternal education, and household economic challenges, highlighting areas for targeted interventions. The DBN model suggests addressing these risk factors early in a child’s life to prevent undernutrition onset or worsening, advocating for policies focused on maternal and child health during critical developmental periods. This tool aids in informed decision-making and improving child health and nutrition in Ethiopia.

## Data Availability

The original contributions presented in the study are included in the article/Supplementary material, further inquiries can be directed to the corresponding author.
