# Supplementary material for: Dynamic Bayesian network modeling for longitudinal data on child undernutrition in Ethiopia (2002–2016)
Source: Front Public Health. 2024 Dec 12;12:1399094. doi: 10.3389/fpubh.2024.1399094 (PMC11669556; doi:10.3389/fpubh.2024.1399094)
Supplement: Supplementary file 1 [file Supplementary_file_1.docx]

**Supplementary Materials for ‘Dynamic Bayesian Network Modeling for Longitudinal Data on Child Undernutrition in Ethiopia (2002-2016)’**

Three intervention programs, PSNP, EAP, and HEP, were initiated at different times: PSNP and EAP started in 2009, while HEP began in 2013. Some households were enrolled in one, two, or all three groups or as a control group (Table S1).

Table S1: Classification and percentage of programme beneficiaries over time in Ethiopia

| Intervention | Description | PSNP | EAP | HEP | 2009 | 2013 | 2016 |
| --- | --- | --- | --- | --- | --- | --- | --- |
| C | Control | No | No | No | 65.0% | 14.0% | 16.0% |
| P | PSNP only | Yes | No | No | 22.0% | 0.4% | 0.2% |
| E | EAP only | No | Yes | No | 9.0% | 1.0% | 0.0% |
| H | HEP only | No | No | Yes | 0.0% | 54.0% | 51.0% |
| PE | PSNP & EAP only | Yes | Yes | No | 4.0% | 0.0% | 0.1% |
| PH | PSNP & HEP only | Yes | No | Yes | 0.0% | 14.0% | 9.0% |
| EH | EAP & HEP only | No | Yes | Yes | 0.0% | 12.0% | 20.0% |
| PEH | PSNP, EAP & HEP | Yes | Yes | Yes | 0.0% | 4.0% | 4.0% |
| PSNP | P+PE+PH+PEH |  |  |  | 26.0% | 19.0% | 13.0% |
| HEP | H+EH+PH+PEH |  |  |  | 0.0% | 85.0% | 83.0% |
| EAP | E+EH+PE+PEH |  |  |  | 13.0% | 18.0% | 24.0% |

*Note: In 2009, H, PH, EH, and PEH were all zero, as HEP was not initiated at that time.*

The study analyzed child undernutrition status using WHO standards and standardized Z scores. As shown in Table S2, the children's anthropometric data were recorded in eight states: normal (N), underweight (U), stunted (S), wasting (W), both underweight and stunted (US), both underweight and wasted (UW), both stunted and wasted (SW), and all underweight, stunted, and wasted.

Table S2: Classification and percentage of child nutritional status over time in Ethiopia

| **Group** | **Description of the group** | **Definition** | **Underweight** | **Stunting** | **Wasting** | **2002** | **2006** | **2009** | **2013** | **2016** |
| --- | --- | --- | --- | --- | --- | --- | --- | --- | --- | --- |
| N | Normal | All WAZ, HAZ, & WHZ>-2SD | No | No | No | 46.2% | 58.1% | 54.1% | 39.6% | 49.9% |
| U | Underweight only | WAZ<-2SD | Yes | No | No | 2.0% | 3.5% | 6.5% | 4.5% | 2.5% |
| S | Stunting only | HAZ<-2SD | No | Yes | No | 16.5% | 15.1% | 4.1% | 1.6% | 1.0% |
| W | Wasting only | WHZ<-2SD | No | No | Yes | 3.2% | 3.0% | 6.8% | 6.6% | 7.3% |
| US | Underweight and stunting | WAZ & HAZ <-2SD | Yes | Yes | No | 18.7% | 14.7% | 13.6% | 13.1% | 10.2% |
| UW | Underweight and wasting | WHZ & WAZ<-2SD | Yes | No | Yes | 6.1% | 4.1% | 11.0% | 20.5% | 14.6% |
| SW | Stunting and wasting | HAZ & WHZ<-2SD | No | Yes | Yes | 0.0% | 0.0% | 0.1% | 0.2% | 0.1% |
| USW | Underweight, stunting, and wasting | All WHZ, WAZ, & HAZ<-2SD | Yes | Yes | Yes | 7.4% | 1.5% | 3.9% | 13.9% | 14.3% |
| Underweight | U+US+UW+USW |  |  |  |  | 34.2% | 23.8% | 35.0% | 52.0% | 41.6% |
| Stunting | S+US+SW+USW |  |  |  |  | 42.6% | 31.3% | 21.7% | 28.8% | 25.6% |
| Wasting | W+UW+SW+USW |  |  |  |  | 16.7% | 8.6% | 21.8% | 41.2% | 36.3% |

Most dataset variables were already categorized, but we applied quantization to continuous variables, including household head age, father’s age, mother’s age, child age, household size, wealth quintiles and the number of antenatal visits. Our approach follows global standards, such as those from the World Health Organization (WHO), and relevant literature, enhancing the model's predictive power. Details of the categorization for all study variables can be found in Table S3 below.

Table S3: Variables in the study and their categorization/ quantization

| No. | Abbreviation | Description | Category (if any) |
| --- | --- | --- | --- |
|  | PS | At least one member is a beneficiary of at least one of the programme (PSNP, EAP, or HEP) in the last 12 months | C, P, E, H, PE, PH, EH, PEH |
|  | CA | Age of the YL child | Infancy, Early childhood, Middle childhood/ Preadolescence, Adolescence |
|  | ME | Mother education level | Illiterate vs Literate |
|  | WQ | Wealth quantile | Poorest, Secondary, Middle, Fourth, Wealthiest |
|  | CS | YL child sex | Male vs Female |
|  | HHA | Household head age | Young, Middle-aged, Elderly |
|  | DA | Father age | Young, Middle-aged, Elderly |
|  | MA | Mother age | Young, Middle-aged, Elderly |
|  | HHS | Household sex | Male vs Female |
|  | DE | Father education level | Illiterate vs Literate |
|  | FS | Household food security status | Sufficient vs Insufficient |
|  | MSW | Mother's subjective wellbeing | Low vs High |
|  | HS | Household size | <=6 vs >6 |
|  | CHI | Child has had serious injury since last round | Yes vs No |
|  | MRH | Mother's relationship with household head | Mother is household head, Mother is partner of household head, and Other |
|  | MLL | Location of YL child's mother | Lives in the household, Does not live in household, and has died |
|  | RSSP | Household has received support from other security programmes in the past 12 months | Yes vs No |
|  | NAV | Number of antenatal visits of mother during pregnancy with YL child | No visits, Low care, Adequate care, Comprehensive care |
|  | BCG | Child have received BCG vaccination | Yes vs No |
|  | HHT | Household head relationship to the YL child | Biological Vs Non-biological |
|  | RG | At least one member got resettled by the government since the previous round | Yes vs No |
|  | DPT | Child have received vaccination against DPT | Yes vs No |
|  | CLHP | Child has long-term health problem | Yes vs No |
|  | CHCP | Child's health compared to peers | Same, Better, Worse |
|  | MEAS | Child has had serious injury/illness since last round | Yes Vs No |
|  | CSW | Children subjective well being | Low vs High |
|  | HIB | Child have received vaccination against HIB | Yes Vs No |
|  | AE | Access to electricity | Yes Vs No |
|  | AC | Access to cooking fuel | Yes Vs No |
|  | ASDW | Access to safe drink water | Yes Vs No |
|  | AT | Access to safe toilet | Yes Vs No |
|  | REG | Region | Tigray, Amhara, Oromiya, SNNP, Addis Ababa C.A |
|  | RESI | Residence | Urban vs Rural |
|  | CHG | General health status of a children | Poor, Average, and Good |
|  | DLL | Location of YL child's father | Lives in the household, Does not live in household, and has died |

**Availability of nodes in the Young Lives dataset by cohort**

The table below displays the availability of nodes across different waves for the young cohort of Ethiopian patients in the Young Lives cohort. White cells indicate missing values, while blue cells indicate missing values.

Table S4: Availability of study variables in the Young Lives dataset by cohort

| **Variables** | **Wave** | | | | |
| --- | --- | --- | --- | --- | --- |
|  | **2002** | **2006** | **2009** | **2013** | **2016** |
| CUS |  |  |  |  |  |
| CA |  |  |  |  |  |
| CS |  |  |  |  |  |
| MSW |  |  |  |  |  |
| MR |  |  |  |  |  |
| HS |  |  |  |  |  |
| WQ |  |  |  |  |  |
| FS |  |  |  |  |  |
| DA |  |  |  |  |  |
| DE |  |  |  |  |  |
| MA |  |  |  |  |  |
| ME |  |  |  |  |  |
| HHA |  |  |  |  |  |
| HHS |  |  |  |  |  |
| PS |  |  |  |  |  |

**Data Preprocessing**

The Young Lives Survey dataset used for this analysis was collected from four countries: Ethiopia, India, Peru, and Vietnam. Research on child health and development typically includes caregiver characteristics as key variables alongside parental characteristics, following a global standard. However, upon a careful review of our original dataset collected from Ethiopia, we found that caregivers are predominantly biological parents (mothers and/or fathers). Specifically, 91.2% of caregivers are biological parents, while the remaining categories consist of non-biological parents (0.9%), grandparents (5.1%), uncles or aunts (1.2%), siblings (0.7%), other relatives (0.7%), and other non-relatives (0.2%), with the roles of partners in-laws (father-in-law/mother-in-law) being virtually nonexistent (0.0%). Additionally, further analysis revealed that caregiver-related variables closely align with parental characteristics. For instance, among caregivers, 92.5% are female, while the remaining 7.5% are male. These findings suggest that the majority of caregivers recorded in this dataset are, in fact, mothers. In Ethiopia, it is a common reality that mothers often serve as the primary caregivers, especially in low-resource households. Consequently, most caregiver-related variables, such as age, education level, and relationship to the household, are already represented by maternal data. Therefore, including caregiver characteristics as independent variables in our analysis does not provide meaningful additional insights, as their information is already captured by parental (mother) data. However, we retained only the subjective well-being of the caregiver as “mother’s subjective well-being,” as all other caregiver variables are effectively captured by maternal data. This refinement allows us to focus our analysis on the relevant parental characteristics.

Subjective wellbeing ratings for mother were collected using a scale ranging from 1 to 9. The wealth quintile is determined by the wealth index, which combines access to durable, service, and housing quality indices. Wealth quintiles are classified as 'poor' for values below 0.6 and 'wealthy' for values above 0.6. The household's food situation was collected under four categories: always eat enough, eat enough but not always, sometimes do not eat enough, and frequently do not eat enough. Furthermore, household size was categorized as either less than 6 or greater than 6 based on the combined number of male and female household members. The mother's relationship with the household was classified as either the household head or the partner. The parents’ levels of education were also categorized as illiterate or literate. The children were classified as infancy (younger than one year), early childhood (1–5 years), middle childhood (6–11 years), or adolescence (12–18 years). Furthermore, the study classified parents, and household heads as young (below 30 years old), middle-aged (30-60 years old), or elderly (over 60 years old).

Due to the extensive number of causal pathways across time points, displaying them all in a single DAG structure has been challenging. Therefore, the detailed DAG causal pathways are presented in Table S5 below. The parent and child nodes, along with the specific time slices where they have causal edges, are indicated by right arrows. These arrows show the temporal direction of the causal connection, specifying which parent node is connected to a child node at which time slice. Additionally, temporal dependency (self-connection) edges are included, indicating where a node has a connection to itself across time slices.

## Fundamental Concepts of Temporal Relationships and Causal Dependencies in DBNs

The nodes, depicted as circles containing data distribution in Figure 2, represent the key variables. These nodes correspond to categorical and continuous variables that have been discretized. In a temporal or DBN, directed edges (arrows) between variables represent statistical or causal dependencies, with direction indicating the nature of these lagged relationships (Figure 2). Each edge specifies a connection between nodes, showing how one variable influences another across the time steps. For example, a link from $X_{t}\text{ to }Y_{t+1}$ designates $X_{t}$ as the parent node at time $t$ and $Y_{t+1}$ as the child node at the subsequent time step. This ordering emphasizes the direction of causality and the dependence of future states on preceding variables, allowing for the analysis of how changes in the parent node's data distribution can affect the child node's distribution over time.

Each node contained the data distribution of its respective variables over time. Each directed edge between nodes represents the strength of the probabilistic relationship between variables across time steps, illustrating the temporal evolution of the dependencies. By utilizing a DBN, it is possible to apply descriptive analytics to explore the relationships among nodes (variables) over time.

In a DBN, temporal links have an order or lag that specifies the time delay between the connected nodes. For instance, a temporal link of order two from node $X_{t}$ to node $Y_{t+2}$ signifies that $X_{t}$ influences $Y_{t+2}$ two time steps in the future; conversely, $Y_{t+2}$ relies on $X_{t}$ 's state two-time steps in the past. While temporal nodes require multiple distributions to account for evolving time-series data, links can also connect non-temporal nodes to temporal nodes, although the reverse is not permitted. Furthermore, if a model includes a temporal link of the order of five, it will incorporate data available up to $t=5$. However, a link with an order of 10 lacks sufficient historical data at this stage, necessitating distinct distributions to handle these differing data availability scenarios effectively.

The relationship between each pair of connected nodes is determined using the Conditional Probability Table (CPT). This table shows the probability strength of the relationship between the data distribution of the parent node and that of the child node (34). The Bayes Server automatically calculated the values in the CPT based on the data distribution of each node in the dataset.

Table S5: Posterior Probabilities and Edge Strengths to identify Potential Relationships in the DAG structure of the DBN Model of Child Undernutrition in Ethiopia (2002–2016)

| **Parent Node** | **Child Node** | **Time 0 to 0** | **Time 0 to 1** | **Time 0 to 2** | **Time 0 to 3** | **Time 0 to 4** | **Time 1 to 1** | **Time 1 to 2** | **Time 1 to 3** | **Time 1 to 4** | **Time 2 to 2** | **Time 2 to 3** | **Time 2 to 4** | **Time 3 to 3** | **Time 3 to 4** | **Time 4 to 4** |
| --- | --- | --- | --- | --- | --- | --- | --- | --- | --- | --- | --- | --- | --- | --- | --- | --- |
| CS | CNS | → 0.53 |  |  |  |  | → 0.15 | → 0.89 |  |  | → 0.10 |  | → 0.89 | → 0.83 |  | → 0.89 |
| CA | CNS | → 0.73 | → 0.61 |  |  |  | → 0.73 | → 0.71 |  |  | → 0.64 | → 0.19 |  | → 0.46 | → 0.27 | → 0.66 |
| MSW | CNS | → 0.83 | → 0.86 | → 0.92 |  |  |  | → 0.77 | → 0.48 |  | → 0.14 | → 0.63 |  | → 0.83 | → 0.81 | → 0.82 |
| FS | CNS |  |  |  |  |  |  |  |  |  | → 0.48 | → 0.41 |  | → 1.00 | → 0.96 | → 1.00 |
| ME | CNS | → 0.83 | → 0.86 |  |  | → 0.72 | → 0.83 |  | → 0.35 |  |  | → 0.76 |  | → 0.87 |  |  |
| DE | CNS | → 0.31 |  |  |  |  | → 0.26 |  |  |  | → 0.48 | → 0.44 |  |  |  | → 0.73 |
| PS | MSW |  |  |  |  |  |  |  |  |  | → 0.74 |  | → 0.79 | → 0.81 | → 0.87 | → 0.93 |
| PS | FS |  |  |  |  |  |  |  |  |  | → 1.00 | → 0.88 | → 0.97 | → 1.00 | → 0.97 | → 1.00 |
| PS | WQ |  |  |  |  |  |  |  |  |  | → 0.82 | → 0.84 | → 0.96 | → 1.00 | → 0.94 | → 1.00 |
| HS | FS |  |  |  |  |  |  |  |  |  | → 1.00 |  |  | → 0.65 | → 0.71 | → 0.89 |
| DE | WQ | → 0.58 |  |  |  |  | → 1.00 | → 0.82 |  |  | → 1.00 |  | → 0.17 | → 1.00 |  | → 0.89 |
| ME | WQ | → 0.93 |  |  |  |  | → 1.00 |  |  |  | → 0.49 |  |  | → 0.58 |  |  |
| DA | WQ | → 0.79 | → 0.63 |  |  |  | → 0.81 |  |  |  | → 1.00 |  | → 0.13 |  | → 0.16 | → 0.90 |
| HHA | WQ | → 0.89 | → 0.71 |  |  |  | → 0.88 | → 0.69 |  |  |  | → 0.18 |  |  | → 0.23 | → 0.22 |
| MA | WQ |  | → 0.18 |  |  |  | → 0.62 | → 0.28 |  | → 0.31 |  |  | → 0.24 |  |  | → 0.58 |
| HHS | WQ | → 0.27 |  | → 0.31 |  |  | → 0.48 | → 0.53 |  |  | → 0.70 | → 0.26 | → 0.18 | → 0.36 | → 0.27 | → 0.72 |
| **Self-connections**  **(temporal dependency)** | | **Time 0 to 1** | **Time 0 to 2** | **Time 0 to 3** | **Time 0 to 4** | **Time 1 to 2** | **Time 1 to 3** | **Time 1 to 4** | **Time 2 to 3** | **Time 2 to 4** | **Time 3 to 4** |  |  |  |  |  |
| CNS | CNS | → 0.94 | → 0.57 |  |  | → 0.63 | → 0.38 |  | → 0.64 |  | → 0.37 |  |  |  |  |  |
| CA | CA | → 0.11 | → 0.21 |  |  | → 0.29 |  |  | → 0.23 |  | → 0.08 |  |  |  |  |  |
| MSW | MSW | → 0.28 | → 0.21 | → 0.07 |  | → 0.17 |  |  | → 0.67 | → 0.79 | → 0.62 |  |  |  |  |  |
| PS | PS |  |  |  |  |  |  |  | → 0.86 | → 0.61 | → 0.96 |  |  |  |  |  |
| FS | FS |  |  |  |  |  |  |  | → 0.92 | → 0.81 | → 0.94 |  |  |  |  |  |
| HS | HS |  | → 0.63 | → 0.44 |  | → 0.13 |  |  | → 0.69 |  |  |  |  |  |  |  |
| DE | DE |  | → 0.37 | → 0.19 |  | → 0.22 |  |  |  | → 0.74 | → 0.18 |  |  |  |  |  |
| ME | ME | → 0.38 |  |  |  | → 0.20 | → 0.26 |  | → 0.28 |  |  |  |  |  |  |  |
| WQ | WQ | → 0.64 | → 0.82 |  |  | → 0.61 | → 0.52 |  | → 0.31 |  | → 0.33 |  |  |  |  |  |
| DA | DA |  | → 0.27 |  |  |  |  | → 0.29 |  | → 0.16 | → 0.31 |  |  |  |  |  |
| HHA | HHA |  |  | → 0.26 |  |  | → 0.38 |  |  |  |  |  |  |  |  |  |
| MA | MA | → 0.62 |  |  |  | → 0.18 |  | → 0.31 |  |  | → 0.27 |  |  |  |  |  |
| HHS | HHS |  |  |  | → 0.31 |  |  | → 0.18 |  | → 0.25 | → 0.15 |  |  |  |  |  |

** NB: A high probability value suggests a strong causal association, while a low probability indicates a weaker association. Values listed as 1.00 represent probabilities close to one, signifying a near-certain relationship but not an exact probability of 1.*

Table S6: Counterfactual predictions of best-case conditions leading to improving child nutrition in Ethiopia

| Variable/ Node | Category | Time slice 0 | | Time slice 1 | | Time slice 2 | | Time slice 3 | | Time slice 4 | |
| --- | --- | --- | --- | --- | --- | --- | --- | --- | --- | --- | --- |
|  |  | Original (%) | Predicted (%) | Original (%) | Predicted (%) | Original (%) | Predicted (%) | Original (%) | Predicted (%) | Original (%) | Predicted (%) |
| Child age | Infancy | 100 | 100 | 0 | 0 | 0 | 0 | 0 | 0 | 0 | 0 |
|  | Early childhood | 0 | 0 | 100 | 100 | 0 | 0 | 0 | 0 | 0 | 0 |
|  | Pre-adolescence | 0 | 0 | 0.3 | 14.9 ↑ | 75.1 | 62.4 ↓ | 24.6 | 22.7 ↓ | 0 | 0 |
|  | Adolescence | 0 | 0 | 0 | 0 | 0 | 0 | 40.9 | 57.2 ↑ | 59.1 | 42.8 ↓ |
| Child sex | Male | 22.8 | 38.1 ↑ | 20.6 | 19.4 ↓ | 20.3 | 13.2 ↓ | 18.2 | 12.6 ↓ | 18.2 | 16.7 ↓ |
|  | Female | 14.4 | 17.3 ↑ | 18.4 | 21.6 ↑ | 18.5 | 16.5 ↓ | 25.9 | 24.8 ↓ | 22.8 | 19.8 ↓ |
| Mother’s Subjective Wellbeing | Low | 35.6 | 28.3 ↓ | 22.9 | 21.4 ↓ | 18.6 | 14.3 ↓ | 12.5 | 11.8 ↓ | 10.5 | 24.2 ↑ |
|  | High | 12.7 | 31.4 ↑ | 18.6 | 19.7 ↑ | 20.6 | 23.1 ↑ | 23.9 | 14.8 ↑ | 24.1 | 11.0 ↓ |
| Household size | <=6 | 22.6 | 31.7 ↑ | 19.7 | 26.2 ↑ | 18.2 | 21.2 ↑ | 20.1 | 17.5 ↓ | 19.5 | 3.4 ↓ |
|  | >6 | 18.4 | 14.6 ↓ | 21 | 17.3 ↓ | 22.9 | 28.3 ↑ | 19.2 | 17.1 ↓ | 18.5 | 22.7 ↑ |
| Wealth Quantile | Poor | 28 | 7.8 ↓ | 2.5 | 1.2 ↓ | 24.2 | 19.3 ↓ | 23.3 | 43.5 ↑ | 21.9 | 28.2 ↑ |
|  | Wealthy | 7.1 | 27.4 ↑ | 16.1 | 31.6 ↑ | 21.2 | 24.8 ↑ | 25.9 | 11.3 ↓ | 29.6 | 4.9 ↓ |
| Food Security status | Insecure | - | - | - | - | 35.5 | 17.1 ↓ | 35.1 | 43.7 ↑ | 29.4 | 39.2 ↑ |
|  | Secure | - | - | - | - | 27 | 38.3 ↑ | 34 | 41.6 ↑ | 39 | 20.1 ↓ |
| Fathe Education Level | Illiterate | 33.6 | 12.7 ↓ | 23.1 | 19.6 ↓ | 21.6 | 19.3 ↓ | 11.2 | 18.7 ↑ | 10.5 | 29.7 ↑ |
|  | Literate | 14.9 | 31.5 ↑ | 18.7 | 26.2 ↑ | 19.1 | 21.8 ↑ | 23.9 | 13.2 ↓ | 23.4 | 7.3 ↓ |
| Mother Education level | Illiterate | 26.9 | 18.3 ↓ | 21.9 | 19.5 ↓ | 19.4 | 16.3 ↓ | 16.2 | 28.4 ↑ | 15.6 | 17.5 ↑ |
|  | Literate | 15.9 | 27.4 ↑ | 18.7 | 26.3 ↑ | 20.3 | 28.1 ↑ | 22.8 | 17.5 ↓ | 22.3 | 0.7 ↓ |
| Father Age | Young | 26.8 | 18.3 ↓ | 24.1 | 27.2 ↑ | 21.1 | 25.8 ↑ | 16.5 | 18.3 ↑ | 11.5 | 10.4 ↓ |
|  | Middle-aged | 24.3 | 19.5 ↓ | 12.2 | 24.2 ↑ | 24.7 | 19.8 ↓ | 23.2 | 13.6 ↓ | 15.7 | 22.9 ↑ |
|  | Elderly | 4.3 | 9.2 ↑ | 8.9 | 8.6 ↓ | 16.3 | 29.5 ↑ | 29.1 | 28.2 ↓ | 41.4 | 24.5 ↓ |
| Mother Age | Young | 35.5 | 31.5 ↓ | 27.8 | 29.8 ↑ | 20.1 | 19.3 ↓ | 11.5 | 12.8 ↑ | 5.1 | 6.6 ↑ |
|  | Middle-aged | 12.7 | 17.4 ↑ | 15.9 | 23.2 ↑ | 20.0 | 18.9 ↓ | 24.5 | 28.3 ↑ | 26.9 | 12.2 ↓ |
|  | Elderly | 7.5 | 9.5 ↑ | 9.2 | 28.1 ↑ | 15.0 | 18.4 ↑ | 29.1 | 27.1 ↓ | 39.2 | 16.9 ↓ |
| Household head Age | Young | 33.3 | 28.3 ↓ | 26.6 | 21.3 ↓ | 19.5 | 19.3 ↓ | 13.4 | 19.3 ↑ | 7.2 | 11.8 ↑ |
|  | Middle-aged | 13.2 | 17.8 ↑ | 16.2 | 26.4 ↑ | 20.0 | 18.3 ↓ | 24.0 | 18.2 ↓ | 26.6 | 19.3 ↓ |
|  | Elderly | 10.6 | 16.3 ↑ | 13.2 | 27.5 ↑ | 21.9 | 19.8 ↓ | 23.8 | 17.1 ↓ | 31.0 | 19.3 ↓ |
| Household head sex | Male | 22.8 | 27.2 ↑ | 20.6 | 21.9 ↑ | 20.3 | 22.4 ↑ | 18.1 | 18.3 ↑ | 18.2 | 10.2 ↓ |
|  | Female | 14.4 | 11.3 ↓ | 18.4 | 11.4 ↓ | 18.5 | 13.5 ↓ | 25.9 | 23.1 ↓ | 22.8 | 40.7 ↓ |

*Note: The dash (-) indicates that the program was unavailable and thus has no edge in the DAG, while zero (0) values signify no recorded data for that time slice.*

Table S7: Counterfactual predictions of worst-case conditions leading to increased child undernutrition in Ethiopia

| Variable/ Node | Category | Time slice 0 | | Time slice 1 | | Time slice 2 | | Time slice 3 | | Time slice 4 | |
| --- | --- | --- | --- | --- | --- | --- | --- | --- | --- | --- | --- |
|  |  | Original (%) | Predicted (%) | Original (%) | Predicted (%) | Original (%) | Predicted (%) | Original (%) | Predicted (%) | Original (%) | Predicted (%) |
| Child age | Infancy | 100 | 100 | 0 | 0 | 0 | 0 | 0 | 0 | 0 | 0 |
|  | Early childhood | 0 | 0 | 100 | 100 | 0 | 0 | 0 | 0 | 0 | 0 |
|  | Pre-adolescence | 0 | 1.0 ↓ | 0.3 | 4.0 ↓ | 75.1 | 65.0 ↓ | 24.6 | 25.5 ↑ | 0 | 5.0 ↑ |
|  | Adolescence | 0 | 0.5 ↑ | 0 | 2.0 ↑ | 0 | 3.0 ↑ | 40.9 | 20.0 ↓ | 59.1 | 70.0 ↑ |
| Child sex | Male | 22.8 | 10.5 ↓ | 20.6 | 15.8 ↓ | 20.3 | 17.1 ↓ | 18.2 | 12.8 ↓ | 18.2 | 43.8 ↑ |
|  | Female | 14.4 | 8.3 ↓ | 18.4 | 25.5 ↑ | 18.5 | 22.3 ↑ | 25.9 | 30.3 ↑ | 22.8 | 13.6 ↓ |
| Mother’s Subjective Wellbeing | Low | 35.6 | 40.2 ↑ | 22.9 | 25.7 ↑ | 18.6 | 15.2 ↓ | 12.5 | 15.8 ↑ | 10.5 | 3.1 ↓ |
|  | High | 12.7 | 5.4 ↓ | 18.6 | 8.3 ↓ | 20.6 | 22.7 ↑ | 23.9 | 40.2 ↑ | 24.1 | 23.4 ↓ |
| Household size | <=6 | 22.6 | 38.2 ↑ | 19.7 | 20.3 ↑ | 18.2 | 12.7 ↓ | 20.1 | 17.3 ↓ | 19.5 | 11.5 ↓ |
|  | >6 | 18.4 | 22.6 ↑ | 21 | 30.4 ↑ | 22.9 | 20.3 ↓ | 19.2 | 16.6 ↓ | 18.5 | 10.1 ↓ |
| Wealth Quantile | Poor | 28 | 42.7 ↑ | 2.5 | 10.4 ↑ | 24.2 | 20.2 ↓ | 23.3 | 18.7 ↓ | 21.9 | 8.0 ↓ |
|  | Wealthy | 7.1 | 3.1 ↓ | 16.1 | 5.7 ↓ | 21.2 | 23.1 ↑ | 25.9 | 32.3 ↑ | 29.6 | 35.8 ↑ |
| Food Security status | Insecure | - | - | - | - | 35.5 | 47.7 ↑ | 35.1 | 41.9 ↑ | 29.4 | 10.4 ↓ |
|  | Secure | - | - | - | - | 27 | 17.1 ↓ | 34 | 23.3 ↓ | 39 | 59.6 ↑ |
| Fathe Education Level | Illiterate | 33.6 | 12.7 ↓ | 23.1 | 19.6 ↓ | 21.6 | 19.3 ↓ | 11.2 | 18.7 ↑ | 10.5 | 29.7 ↑ |
|  | Literate | 14.9 | 31.5 ↑ | 18.7 | 26.2 ↑ | 19.1 | 21.8 ↑ | 23.9 | 13.2 ↓ | 23.4 | 7.3 ↓ |
| Mother Education level | Illiterate | 26.9 | 18.3 ↓ | 21.9 | 19.5 ↓ | 19.4 | 16.3 ↓ | 16.2 | 28.4 ↑ | 15.6 | 17.5 ↑ |
|  | Literate | 15.9 | 27.4 ↑ | 18.7 | 26.3 ↑ | 20.3 | 28.1 ↑ | 22.8 | 17.5 ↓ | 22.3 | 0.7 ↓ |
| Father Age | Young | 26.8 | 18.3 ↓ | 24.1 | 27.2 ↑ | 21.1 | 25.8 ↑ | 16.5 | 18.3 ↑ | 11.5 | 10.4 ↓ |
|  | Middle-aged | 24.3 | 19.5 ↓ | 12.2 | 24.2 ↑ | 24.7 | 19.8 ↓ | 23.2 | 13.6 ↓ | 15.7 | 22.9 ↑ |
|  | Elderly | 4.3 | 9.2 ↑ | 8.9 | 8.6 ↓ | 16.3 | 29.5 ↑ | 29.1 | 28.2 ↓ | 41.4 | 24.5 ↓ |
| Mother Age | Young | 35.5 | 31.5 ↓ | 27.8 | 29.8 ↑ | 20.1 | 19.3 ↓ | 11.5 | 12.8 ↑ | 5.1 | 6.6 ↑ |
|  | Middle-aged | 12.7 | 17.4 ↑ | 15.9 | 23.2 ↑ | 20.0 | 18.9 ↓ | 24.5 | 28.3 ↑ | 26.9 | 12.2 ↓ |
|  | Elderly | 7.5 | 9.5 ↑ | 9.2 | 28.1 ↑ | 15.0 | 18.4 ↑ | 29.1 | 27.1 ↓ | 39.2 | 16.9 ↓ |
| Household head Age | Young | 33.3 | 28.3 ↓ | 26.6 | 21.3 ↓ | 19.5 | 19.3 ↓ | 13.4 | 19.3 ↑ | 7.2 | 11.8 ↑ |
|  | Middle-aged | 13.2 | 17.8 ↑ | 16.2 | 26.4 ↑ | 20.0 | 18.3 ↓ | 24.0 | 18.2 ↓ | 26.6 | 19.3 ↓ |
|  | Elderly | 10.6 | 16.3 ↑ | 13.2 | 27.5 ↑ | 21.9 | 19.8 ↓ | 23.8 | 17.1 ↓ | 31.0 | 19.3 ↓ |
| Household head sex | Male | 22.8 | 18.3 ↓ | 20.6 | 27.2 ↑ | 20.3 | 21.9 ↑ | 18.1 | 22.4 ↑ | 18.2 | 10.2 ↓ |
|  | Female | 14.4 | 21.3 ↑ | 18.4 | 21.4 ↑ | 18.5 | 23.5 ↑ | 25.9 | 27.1 ↑ | 22.8 | 6.7 ↓ |

*Note: The dash (-) indicates the program was unavailable, and zero (0) values indicate no recorded value for that time slice.*
